# Bacterial Cellulose Nanocrystal-Stabilized Water-in-Water Pickering Emulsions: Stability, Amylopectin Partitioning, and In Vitro Digestion Behavior

**DOI:** 10.3390/foods15142550

**Published:** 2026-07-20

**Authors:** Yuhan Jiang, Sha Ao, Xianwen Hu, Shilin Liu

**Affiliations:** 1 College of Chemistry, Huazhong Agricultural University, Wuhan 430070, China; wy5857549@163.com; 2 College of Food Science & Technology, Huazhong Agricultural University, Wuhan 430070, China; slliu2013@mail.hzau.edu.cn

**Keywords:** aqueous two-phase systems, bacterial cellulose, stability, starch digestion

## Abstract

Water-in-water (W/W) Pickering emulsions based on aqueous two-phase systems (ATPS) are promising for food applications, but their stabilization is challenged by the ultra-low interfacial tension inherent to ATPSs. This study developed food-grade W/W Pickering emulsions stabilized by bacterial cellulose nanocrystals (BCNCs) and evaluated their ability to regulate starch digestibility. The effects of dextran (Dex) concentration, maltodextrin (MD) concentration, and BCNC content on the microstructure, rheological properties, and stability of the emulsions were systematically investigated. The partitioning behavior of amylopectin (AMP) between the two phases and the in vitro digestion behavior of AMP-loaded W/W Pickering emulsions in the presence of α-amylase were further examined. Fourier-transform infrared spectroscopy confirmed the successful preparation of BCNCs. The optimal formulation (24 wt% Dex, 14 wt% MD, and 0.24 wt% BCNCs) exhibited a uniform droplet distribution and enhanced storage stability. Contact angle analysis indicated preferential wettability of BCNCs toward the continuous Dex-rich phase, promoting interfacial adsorption and droplet stabilization. The emulsions remained stable at pH 3.0–7.0 but were sensitive to ionic strength above 7 mM sodium chloride. Importantly, in vitro digestion showed that BCNC-stabilized emulsions significantly inhibited α-amylase-mediated hydrolysis of AMP, with the hydrolysis extent reducing from ~54% to 14%. These results indicate that BCNCs act as effective natural stabilizers for W/W Pickering emulsions, forming a physical barrier that retards starch digestion. This work provides a sustainable strategy for designing slow-digestible, low-glycemic-index food systems with potential applications in functional foods and pharmaceuticals.

## 1. Introduction

Aqueous two-phase systems (ATPSs) are biphasic systems formed when aqueous solutions of mutually immiscible hydrophilic polymers, polymer–salt combinations, or salt–salt mixtures exceed a critical concentration leading to spontaneous phase separation. Owing to their excellent biocompatibility, eco-friendliness, and tunable properties, ATPSs have been widely applied in food processing, biomedicine, tissue engineering, cell separation, and green chemical extraction [1]. Unlike conventional emulsions stabilized by molecular surfactants, Pickering emulsions are stabilized by solid particles that adsorb irreversibly at the interface, forming a mechanical barrier around droplets and thereby effectively suppressing coalescence and improving emulsion stability. As an extension of ATPSs, W/W Pickering emulsions employ solid particles to stabilize the interface between immiscible aqueous phases [2]. It is worth noting that the adsorption energy of particles at the interface is directly proportional to the interfacial tension. Therefore, the ultra-low interfacial tension characteristic of ATPSs significantly weakens the driving forces for particle adsorption, posing a fundamental challenge for emulsion stabilization [3,4]. Consequently, a pivotal focus in current research is the development of colloidal particles capable of enhancing interfacial anchoring to enable the fabrication of high-performance W/W Pickering emulsions.

To address this limitation, oppositely charged polyelectrolytes have been explored to enhance interfacial stability via complexation at the interface [5]. However, most reported systems rely on synthetic polymers, such as the polyanions poly(acrylic acid), poly(sodium-4-styrene sulfonate), polycations poly(ethylene imine), and poly(diallyldimethylammonium chloride) [6,7], which restrict their applications in food-grade systems. Natural biopolymers, including polysaccharides [8] and proteins [9], have therefore gained increasing attention as stabilizers due to their biocompatibility and biodegradability, offering more favorable alternatives for stabilizing ATPS-based emulsion systems. Although inorganic particles have been extensively studied for stabilizing W/W Pickering emulsions [10,11], their limited biodegradability and biocompatibility hindered their practical application in safety-sensitive fields such as food and pharmaceuticals.

Bacterial cellulose (BC) is a natural polysaccharide synthesized by microorganisms and is known for its outstanding mechanical properties, biocompatibility, and renewability. Although inherently water-insoluble, BC can be converted into nanoscale materials, such as bacterial cellulose nanofibers (BCNF) or bacterial cellulose nanocrystals (BCNCs), through chemical or a combination of chemical and mechanical treatments [12]. These processes reduce particle size while introducing surface charges, enabling aqueous dispersibility. To date, research on cellulose nanocrystals (CNCs) from plants as stabilizers has primarily focused on oil–water Pickering emulsions. For example, Bai et al. [13] fabricated CNCs via sulfuric acid hydrolysis and successfully stabilized water-in-oil emulsions using high-energy microfluidization. Varanasi et al. [14] demonstrated that CNCs could stabilize emulsions regardless of surface charge density. Chen et al. [15] enhanced CNCs’ hydrophobicity through octenyl succinic anhydride esterification, enabling the stabilization of high internal phase emulsions, while other studies reported the development of amphiphilic CNCs with improved emulsifying efficiency and hydrophobic modification [16]. In recent years, CNCs have also received increasing attention for stabilizing W/W Pickering emulsions. Peddireddy et al. [17] reported that CNC-stabilized W/W Pickering emulsions in a polyethylene oxide/dextran system exhibited weak gel formation in the presence of sodium chloride, effectively preventing phase separation. Nie et al. [18] further demonstrated that increasing CNC concentration significantly improved the storage stability of W/W Pickering emulsions. Additional studies have also explored CNC-stabilized all-aqueous systems [8,19,20,21,22,23,24]. Given their excellent biocompatibility, biodegradability, renewability, non-toxicity, and strong interfacial adsorption capacity, BCNCs show great promise for stabilizing high-performance W/W Pickering emulsions.

In this work, food-grade W/W emulsions were constructed using a Dex/MD-based ATPSs stabilized by BCNCs. The effect of polymer concentration and BCNC content on emulsion microstructure and stability was comprehensively examined. Furthermore, the influence of AMP on emulsion morphology, interfacial structure, and partitioning behavior of AMP was elucidated, along with its enzymatic hydrolysis behavior in the emulsion system. This study not only provides a systematic evaluation of BCNC-stabilized Dex/MD-based W/W Pickering emulsions, but also establishes a link between emulsion structure and functional molecule behavior, offering new insights into the design of sustainable, food-grade all-aqueous delivery systems with controlled digestion characteristics.

## 2. Materials and Methods

### 2.1. Materials

Dextran (Dex), amylopectin (AMP), potassium bromide (spectral grade), fluorescein isothiocyanate-labeled Dextran (FITC-Dex), and FITC (analytical grade) were purchased from Aladdin Chemical Reagents Co., Ltd. (Shanghai, China). Maltodextrin (MD) was kindly provided by Zhejiang NHU Co., Ltd. (Shaoxing, China). Bacterial cellulose was purchased from Hainan Yida Food Co., Ltd. (Haikou, China). The α-amylase (from hog pancreas, ~50 U/mg) was purchased from Sigma-Aldrich, Merck KGaA (Darmstadt, Germany). Hydrochloric acid, sodium hydroxide, concentrated sulfuric acid, and sodium chloride were all of analytical grade and acquired from Sinopharm Chemical Reagent Co., Ltd. (Shanghai, China).

### 2.2. Preparation of Bacterial Cellulose Nanocrystals

Inorganic strong acid hydrolysis is one of the most established and widely used methods for producing BCNCs [25]. This technique relies on the principle that hydrogen ions (H^+^) preferentially attack the amorphous regions of cellulose, cleaving glycosidic bonds and releasing nanocrystals with high crystallinity. BCNCs were prepared from BC using a two-step process consisting of mechanical homogenization followed by sulfuric acid hydrolysis [12]. Initially, the BC was thoroughly rinsed with distilled water until the washing reached a neutral pH. It was then pre-coarsely shredded using a juicer (BL806A, Zhongshan, China) to process a crude BC suspension. This suspension was further processed using a high-pressure homogenizer (AH-1500, Shanghai, China) at 600 bar for 10 cycles to obtain BCNF. The BCNF was concentrated using a rotary evaporator, dried, and stored at 4 °C for later use. For acid hydrolysis, 4 g dry weight of BCNF was dispersed in 100 mL of 64% sulfuric acid and stirred with a water bath at 45 °C for 90 min using a magnetic stirrer. The reaction was quenched by adding distilled water at five times the reaction volume. The resulting suspension was centrifuged at 10,000 rpm for 20 min, and the supernatant was discarded. The washing step was repeated with distilled water until the pH reached neutrality, followed by additional centrifugation. To remove any residual acid and low-molecular-weight impurities, the BCNCs were dialyzed in a 3000 Da molecular cut-off dialysis membrane for 48 h. Finally, the dialyzed suspension was concentrated using a rotary evaporator (GC03, Shanghai, China), vacuum-dried, and weighed to determine the final mass fraction. The BCNCs were stored at 4 °C for future use.

### 2.3. Preparation of W/W Pickering Emulsions

Prior to the preparation of W/W Pickering emulsions, 50 wt% stock solutions of Dex and MD were prepared, respectively. A predetermined volume of Dex and MD stock solutions was mixed and homogenized using a high-speed shear mixer (T18, Shanghai, China) at 11,000 rpm for 2 min to obtain a W/W emulsion. Subsequently, BCNCs at desired concentrations were added to the W/W emulsion, followed by magnetic stirring at 400 rpm for 10 min to obtain the final W/W Pickering emulsions. The effects of Dex concentration (18, 20, 22, and 24 wt%), MD concentration (8, 10, 12, and 14 wt%), and BCNC concentration (0.16, 0.20, 0.24, and 0.28 wt%) on the microstructure and stability of the W/W emulsions were systematically investigated.

### 2.4. Micromorphology of BCNCs

The morphology of BC and BCNCs was characterized using atomic force microscopy (AFM, MultiMode 8, Bruker, Billerica, MA, USA). Each sample was first prepared as a 0.005 wt% suspension. A 10 μL aliquot of the suspension was dropped onto the center of a freshly cleaved mica sheet and allowed to air-dry at ambient temperature for 3 h. Once dried, the mica substrates were mounted onto the AFM stage, and imaging was performed in tapping mode. The acquired images were analyzed using NanoScope Analysis software (Version 1.9, Veeco, Santa Barbara, CA, USA), which was used to extract and calculate the average width and height of BC and BCNCs, allowing for quantitative comparison of their morphological features.

### 2.5. Fourier-Transform Infrared (FT-IR) Spectroscopy

Powdered samples of BC and BCNCs were obtained by lyophilization using a freeze dryer (BT48, Millrock Technology, Kingston, NY, USA). The structures of these samples were characterized using Fourier transform infrared spectroscopy (FTIR-ATR, Nexus-470, Nicolet, Madison, WI, USA). Firstly, an accurately weighed sample was mixed with potassium bromide in a mass ratio of 1:100. Then, a transparent pellet was fabricated utilizing a tablet press. Finally, the infrared spectrum was recorded over the range of 4000 to 400 cm^−1^ at a resolution of 2 cm^−1^, with an average of 64 scans per sample.

### 2.6. ζ-Potential

The BCNC suspension was diluted to a concentration of 0.1 wt%, and its pH was adjusted to a range of 2 to 12 using 1 M hydrochloric acid (HCl) or 1 M sodium hydroxide (NaOH). ζ-potential measurements were performed at 25 °C using a Zetasizer Nano instrument (Malvern Instruments, Malvern, UK). To evaluate the effect of ionic strength, varying amounts of NaCl were added to the suspension to achieve final concentrations ranging from 0 to 15 mM, followed by additional ζ-potential measurements. Each sample was measured in triplicate, and the mean value and standard deviation were calculated.

### 2.7. Contact Angle

The static contact angle was measured using a contact angle goniometer (OCA15EC, Dataphysics, Filderstadt, Germany). Freeze-dried BC and BCNC powders were compressed into flat, dense pellets under 25 MPa pressure for 30 s to serve as solid substrates. Aqueous solutions of MD (14 wt%) and Dex (24 wt%), prepared at the same concentrations used in the emulsion formulations, were employed as probe liquids. A 2 µL droplet of each liquid was carefully placed onto the sample surface using a motorized microsyringe (SNS 021/011). The stage was adjusted to allow immediate contact between the droplet and substrate. The droplet profile was continuously recorded by the built-in high-resolution camera of the OCA15EC instrument, and the contact angle was calculated using SCA20 software based on the Laplace-Young fitting method. Each sample was measured at least six times, and the average value and standard deviation were reported to ensure data reliability.

### 2.8. Emulsion Microstructure

The microstructure of W/W Pickering emulsions was observed using an optical microscope (EX31, SDPTOP Optical Microscope, Ningbo, China) equipped with a 40× objective lens, and representative images were captured. The average droplet size was quantified using *ImageJ* software (ImageJ 1.54f) by measuring at least 300 droplets (*n* = 300). To further visualize the distribution of the Dex-rich phase, FITC-Dex was used to stain the Dex-rich domains. Fluorescence microscopy (DM3000, Leica Microsystems, Wetzlar, Germany) with a 40× objective lens was employed to observe the labeled structures.

### 2.9. Storage and Stress Stability of the Emulsions

To investigate the storage stability of W/W Pickering emulsions, 6 mL of freshly prepared emulsion was transferred into 10 mL glass vials and stored at ambient temperature. To evaluate the effects of pH and ionic strength, stock solutions of 100 mM NaCl and 1 M HCl or NaOH were prepared. Appropriate volumes of NaCl solution were added to the emulsion to achieve final NaCl concentrations of 0, 1, 3, 5, and 7 mM. Similarly, pH adjustments were made by HCl or NaOH solutions to set the emulsion pH between 3 and 11. All samples were stored at ambient temperature, and their microstructure and macroscopic phase separation were monitored at 0 h, day 3, and day 5 to evaluate storage, ionic strength, and pH stability.

### 2.10. Rheological Properties

The rheological properties of freshly prepared emulsions were measured using a rotational rheometer (HR-2, TA Instruments, New Castle, DE, USA) equipped with a 60 mm cone plate and a cone angle of 1.007°. The emulsion sample was evenly loaded into the gap between the cone and plate; steady shear measurements were performed over a shear rate range of 0.1 to 100 s^−1^, and the viscosity (η) as a function of shear rate (γ) was recorded in real time.

### 2.11. In Vitro Digestion

In vitro digestion experiments were conducted according to the method reported by Zhang and Nie et al. [18,26] with minor modifications. In all samples, BCNC, Dex, and MD were used at their optimized concentrations of 0.24 wt%, 24 wt%, and 14 wt%, respectively, and the total mass of each system was fixed at 10 g. Six groups of samples were prepared as follows: (1) a pure amylopectin (AMP) solution at 0.6 wt% (denoted as 0.6AMP); (2) a Dex/MD system containing 0.6 wt% AMP without stabilizer (Dex/MD-0.6AMP); a BCNC-stabilized Dex/MD Pickering emulsion containing (3) 0.2 wt%, (4) 0.4 wt%, (5) 0.6 wt%, (6) and 0.8 wt% AMP (Dex/MD-BCNC-0.2/0.4/0.6/0.8AMP). All samples were pre-incubated at 37 °C for 30 min. Subsequently, 2 mL of phosphate-buffered solution (0.02 mol/L, pH 6.9) containing 3 mmol/L calcium chloride (CaCl_2_) and 500 U of α-amylase was added to each sample. The digestion reaction was carried out at 37 °C with continuous shaking at 160 rpm for 4 h. During digestion, 0.5 mL aliquots were withdrawn at predetermined time points (0, 5, 10, 20, 30, 60, 120, and 240 min) and immediately mixed with 2 mL of anhydrous ethanol to terminate enzyme activity. The mixtures were centrifuged at 5000 rpm for 5 min, and the supernatants were collected. The reducing sugar content in the supernatant was determined using the 3,5-dinitrosalicylic acid (DNS) method, with glucose used as the standard. The hydrolysis percentage of the samples was then calculated. The digestion kinetics at different time points were fitted using a pseudo-first-order kinetic model, expressed as:

$$p_{t}=p_{\infty }\times (1-\exp\left(-kt\right))$$

where $p_{t}$ is the hydrolysis percentage at time *t*, $p_{\infty }$ is the hydrolysis percentage at the endpoint, and k is the pseudo-first-order rate constant.

### 2.12. Statistical Analysis

All experiments were independently conducted at least three times. Results were expressed as the mean ± standard deviation (SD). Data processing and statistical analyses were performed using Origin 2024 (Student Version) and IBM SPSS 25 software, with Photoshop 2019 being used for image processing. Significant differences between samples were determined by Duncan’s test at a significance level of *p* < 0.05.

## 3. Results and Discussion

### 3.1. Micromorphology and Characterization of BCNCs

**Figure 1:**
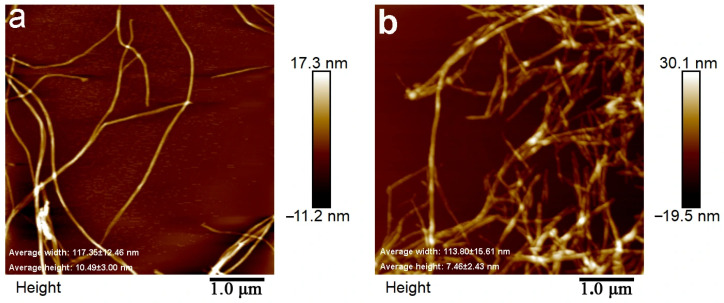
Atomic force micrographs of (**a**) BC and (**b**) BCNCs.

The morphological characteristics of BC before and after hydrolysis were first investigated using AFM. As shown in Figure 1a, both homogenized BC and hydrolyzed BCNCs exhibited a loose and interconnected fibrous network structure. After sulfuric acid hydrolysis, partial degradation of amorphous domains occurred, leading to a reduction in fiber integrity and promoting fragmentation of cellulose fibrils. Simultaneously, the increased specific surface area raised the surface energy, which in turn induced noticeable aggregation and overlap among BCNC fibrils (Figure 1b), consistent with previously reported results [12]. Quantitative analysis using NanoScope Analysis software further revealed that the untreated BC had an average width of 117 ± 12 nm and a height of 10 ± 3 nm. After hydrolysis, the BCNCs exhibited slightly reduced dimensions, with an average width of 113 ± 15 nm and a height of 7 ± 2 nm. However, due to severe entanglement of fibrils, reliable measurement of fibril length was not feasible. The treatment of BC with sulfuric acid for a relatively short duration had minimal impact on its size but a greater change in aspect ratio. Overall, the combined treatment of high-pressure homogenization and acid hydrolysis resulted in a modest reduction in lateral dimensions and aspect, which is beneficial for achieving a more uniformly dispersed suspension.

**Figure 2:**
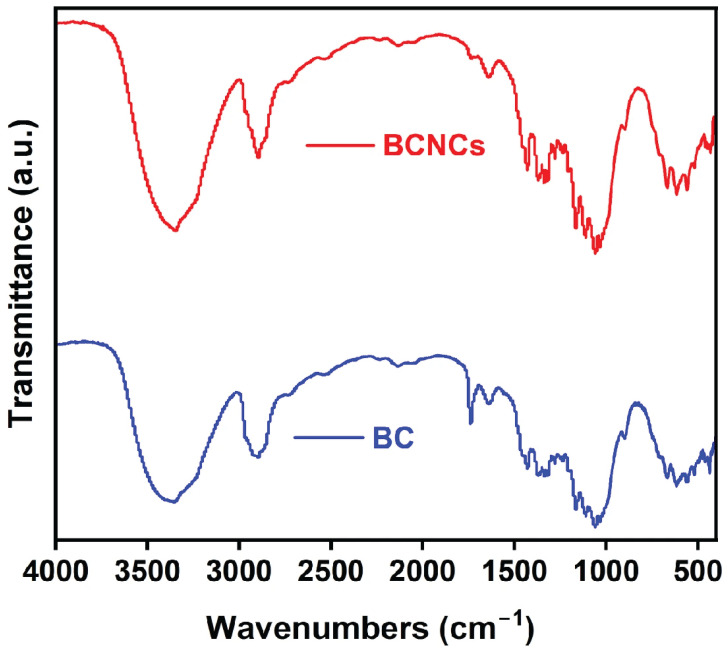
FT-IR spectra of BC and BCNCs.

The chemical structure of the BCNCs was confirmed by FT-IR spectroscopy, with a comparative analysis against native BC. As illustrated in Figure 2, both BC and BCNCs exhibited a characteristic absorption band around 1640 cm^−1^, attributed to -OH groups from water molecules. This peak reflected strong hydrogen bonding between cellulose chains and water, making complete moisture removal challenging [27]. A broad band at approximately 3350 cm^−1^ corresponded to the stretching vibrations of -OH groups [28]. Peaks near 2900 cm^−1^ and 1430 cm^−1^ are assigned to C-H bond stretching and asymmetric bending vibrations of methylene and methyl groups, respectively. Additional peaks at 1371 cm^−1^, 1111 cm^−1^, and 1059 cm^−1^ could be ascribed to the symmetric bending of C-H bonds and to C-C-OH and C-OH stretching vibrations in secondary and primary alcohols, respectively [27,29]. The band at 898 cm^−1^ corresponded to the asymmetric stretching vibration of the C-O-C bond [30]. These absorption bands represented the characteristic vibrations of the BC backbone [31]. In this study, the characteristic peaks were observed at 1430 and 898 cm^−1^. Taken together, these findings confirm that BCNCs were successfully prepared via sulfuric acid hydrolysis.

### 3.2. ζ-Potential

**Figure 3:**
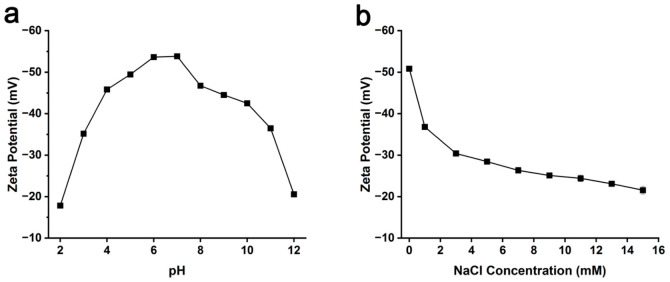
Effects of varying (**a**) pH (2–12) and (**b**) NaCl (0–15 mM) concentration on the ζ-potential of BCNC suspensions.

Apparent ζ-potential serves as a key indicator for evaluating the surface charge characteristics and dispersion stability of colloidal systems. Typically, when the absolute value of the apparent ζ-potential exceeds 30 mV, the system is considered highly stable [32,33]. The stability of BCNC suspensions was found to be strongly influenced by both pH and ionic strength. As shown in Figure 3a, the apparent ζ-potential was shifted from −12 ± 0.9 mV to −53 ± 0.2 mV when increasing the pH from 2 to 12. Across the entire pH range, consistently negative values were observed, primarily due to two factors. The absence of cationic functional groups on the BCNCs and the presence of the negatively charged -OSO_3_^−^ group introduced via sulfuric acid-mediated esterification of surface hydroxyls [12], which resulted in negative surface charge. By comparison, nanocrystals prepared using hydrochloric acid generally exhibit negligible surface charge [27]. Further analysis revealed a non-monotonic relationship between apparent ζ-potential and pH. From pH 2 to 7, gradual deprotonation of the -SO_3_^−^ groups led to a sharp increase in the absolute apparent ζ-potential, from −17 ± 0.4 mV to −53 ± 0.2 mV. However, at higher pH values, enhanced electrostatic screening within the solution reduced the effective surface charge, resulting in a slight decline in the absolute apparent ζ-potential. Notably, at pH 2 the apparent ζ-potential (−17 ± 0.4 mV) was below the threshold required for colloidal stability, and pronounced aggregation was observed. In addition, substantial variations in the apparent ζ-potential were observed when the pH was adjusted within the range of 4–8, particularly under neutral conditions. Given that the pKa of the sulfate half-ester groups on CNCs is approximately 1.9, indicating that these groups are fully dissociated at pH values above 3, the observed pH-dependent changes imply the possible presence of additional surface functionalities or impurities on the BCNCs used in this study, such as carboxyl groups. To improve the accuracy and reproducibility of ζ-potential measurements, future work may incorporate the addition of 5–10 mM electrolytes (e.g., NaCl) to minimize polarization effects and charge-screening artifacts.

The influence of NaCl concentration on the apparent ζ-potential of BCNC suspensions was also investigated over an NaCl concentration range of 0–15 mM. As shown in Figure 3b, the apparent ζ-potential decreased significantly from −50 ± 0.2 mV to −21 ± 0.9 mV as NaCl concentration was increased. This reduction was primarily attributed to the compression of the electrical double layer and the electrostatic shielding by oppositely charged sodium ions, which weaken inter-particle repulsion and lower the absolute apparent ζ-potential. A similar trend was previously reported by Winuprasith and his-co-workers [34]. It was noteworthy that at low ionic strength, sodium ions could enhance the energy barrier between particles, thus effectively inhibiting flocculation or coalescence. However, once the ion concentration exceeded a critical threshold, this energy barrier collapsed, leading to charge neutralization and particle aggregation, which resulted in a notable loss of colloidal stability. Furthermore, further increases in sodium ion concentration continued to reduce the surface charge density of the system, severely weakening electrostatic stabilization, and ultimately inducing irreversible aggregation. It should be emphasized that, owing to the anisotropic rod-like morphology of BCNCs, the ζ-potentials reported herein are apparent values derived from the Smoluchowski approximation and are intended solely for comparative purposes; they do not represent the exact ζ-potential. To quantify the surface charge of anisotropic, high-aspect-ratio BCNCs with greater accuracy, advanced models such as anisotropic electrophoresis theory or the methodology detailed by Pirich et al. [35].

### 3.3. Influence of MD Concentration on W/W Pickering Emulsions

**Figure 4:**
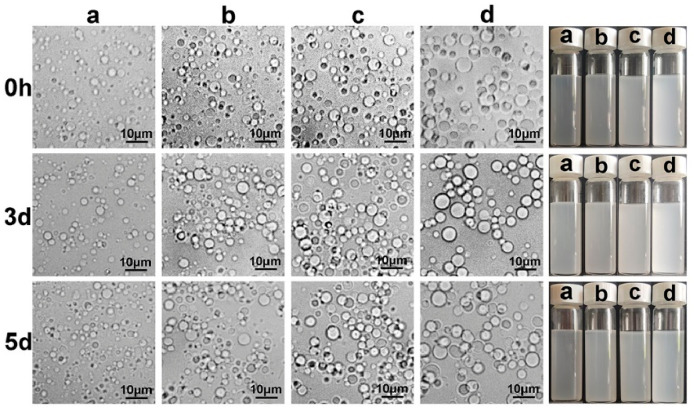
Effects of MD concentration on the droplet structure and macroscopic phase separation of W/W Pickering emulsions after 5 days of storage at ambient temperature: (**a**) 8 wt%, (**b**) 10 wt%, (**c**) 12 wt%, and (**d**) 14 wt%. The concentrations of BCNCs and Dex were fixed at 0.16 wt% and 20 wt%, respectively. Scale bar: 10 μm.

In preliminary work [24], the phase diagram (Figure S1), phase separation behavior, and emulsification parameters of the Dex/MD ATPS in the W/W system were systematically investigated. Based on these results and guided by additional pre-experiments, the Dex and BCNC concentrations were fixed at 20 wt% and 0.16 wt%, respectively. The effects of varying MD concentrations on the stability and rheological properties of W/W Pickering emulsions were then examined. As shown in Figure 4 and Table S1, the average droplet size increased from 2.3 ± 0.3 μm to 4.0 ± 0.5 μm as the MD concentration increased from 8 wt% to 14 wt% (*p* < 0.05). This phenomenon can be attributed to the fixed amount of BCNCs, which limited the total interfacial area that could be effectively covered. As the volume fraction of the internal phase increased, the insufficient surface led to droplet coalescence or aggregation, resulting in larger droplets. This mechanism resembles that observed in traditional oil–water systems, where the average droplet size increased with the oil phase proportion [36]. Optical microscopy revealed that all droplets were spherical or slightly elliptical, with no signs of rupture or significant aggregation. Optical microscopy tracking over 5 days showed no significant changes in droplet size across all formulations. However, macroscopic observation revealed a thin, clear layer at the top of the emulsion containing 8 wt%, 10 wt%, and 12 wt% MD, indicating slight phase separation. This phenomenon was not observed in the 14 wt% MD emulsion. The improved stability was likely due to increased viscosity of the emulsion and the interconnected fibrous network of BCNCs in the system phase, which reduced droplet Brownian motion and enhanced molecular entanglement, forming a weak network that counteracted gravitational sedimentation and suppressed interfacial fluctuations.

**Figure 5:**
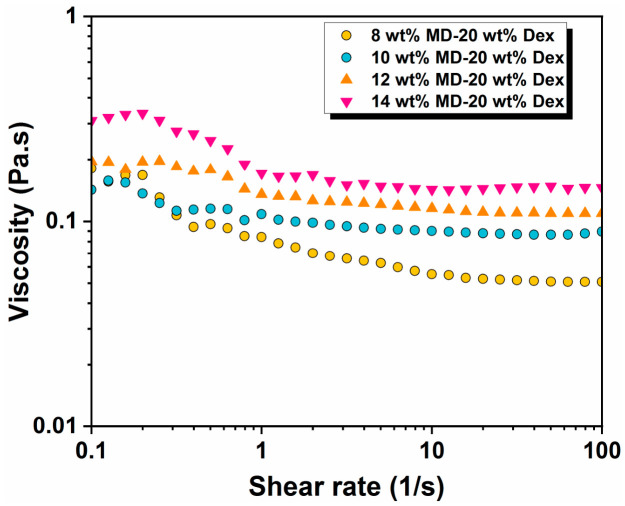
Relationship between viscosity and shear rate of W/W Pickering emulsions constructed with different concentrations of MD. The concentrations of BCNCs and Dex were fixed at 0.16 wt% and 20 wt%, respectively.

To further verify these results, the rheological behavior of the emulsions was studied under constant BCNC concentration (0.16 wt%) and Dex concentration (20 wt%), with only the MD concentration being varied. Figure 5 illustrates the relationship between the viscosity of the BCNC-stabilized W/W Pickering emulsions and the shear rate. It was found that all emulsions exhibited typical shear-thinning behavior, with viscosity decreasing as the shear rate increased, characteristic of non-Newtonian fluids. This is attributed to the increase in the total polysaccharide content with rising MD concentration, which enhances chain entanglement and intermolecular hydrogen bonding, thereby increasing the viscosity of the emulsions [37]. Under the same shear rate conditions, the viscosity of the emulsions significantly increased with high MD concentrations. As pointed out by Tea et al. [38], for W/W emulsions, the shear-thinning behavior of the continuous phase is crucial, but the shear-thinning of the dispersed phase also affects the viscosity of the emulsions. In previous studies, it was found that the viscosity of W/W emulsions changes with the shear rate, to some extent reflecting the relation of the dispersed droplets, and changes in droplet size cause shifts in characteristic frequencies or relaxation times [39]. Furthermore, the flow curve exhibited a “two-stage” profile, with a Newtonian plateau observed at both very low (<0.01 s^−1^) and very high (>100 s^−1^) shear rates [40]. This Newtonian behavior has been attributed to the dynamic balance between polysaccharide entanglement–disentanglement and network breakdown–reformation under shear stress [41]. Therefore, the rheological behavior of these emulsions was a result of the combined effects of MD, BCNC interactions and droplets under different shear conditions.

### 3.4. Influence of Dex Concentration on W/W Pickering Emulsions

**Figure 6:**
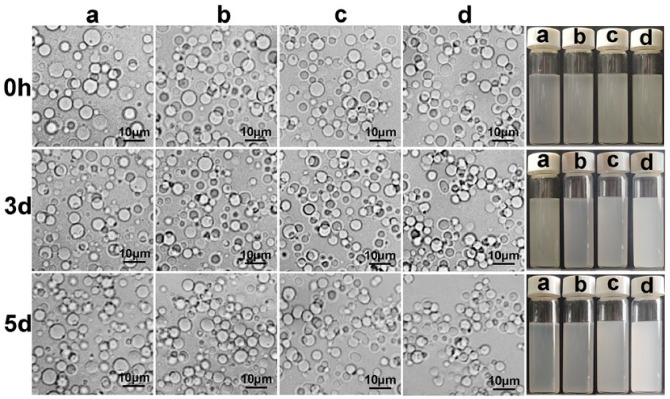
Effects of Dex concentration on the droplet structure and macroscopic phase separation of W/W Pickering emulsions after 5 days of storage at ambient temperature: (**a**) 18 wt%, (**b**) 20 wt%, (**c**) 22 wt%, and (**d**) 24 wt%. The concentrations of BCNCs and MD were fixed at 0.16 wt% and 14 wt%, respectively. Scale bar: 10 μm.

Building on the previous findings regarding the effects of MD concentration on average droplet size and emulsion stability, the variables were further refined by fixing the concentration of BCNCs (0.16 wt%) and MD (14 wt%), while systematically varying only the Dex concentration (18–24 wt%) to investigate the influence of continuous phase composition on the microstructure, storage behavior, and rheological properties of W/W Pickering emulsions. Optical microscopy observations revealed that increasing the Dex concentration, from 18 wt% to 24 wt% led to a decrease in average droplet size from 4.6 ± 0.7 μm to 3.2 ± 0.5 μm (Figure 6 and Table S2). This trend can be attributed to two factors. First, with a constant stabilizer concentration, increasing the Dex concentration reduced the volume ratio of dispersed phase to continuous phase. This change effectively increased the available BCNC coverage per unit interfacial area, thereby suppressing droplet coalescence. Second, the rise in Dex concentration significantly enhanced the viscosity of the continuous phase, which helped prevent flocculation and coalescence-driven phase separation [42]. Over a 5-day storage period, no visible macroscopic phase separation was observed in any of the emulsions. Slight flocculation was noted only under high magnification in samples containing 24 wt% Dex. In an all-aqueous system, although Ostwald ripening is not typically a concern, flocculation and coalescence remain the primary mechanisms of destabilization [43]. Among the tested formulations, the emulsion containing 24 wt% Dex exhibited the best physical stability.

**Figure 7:**
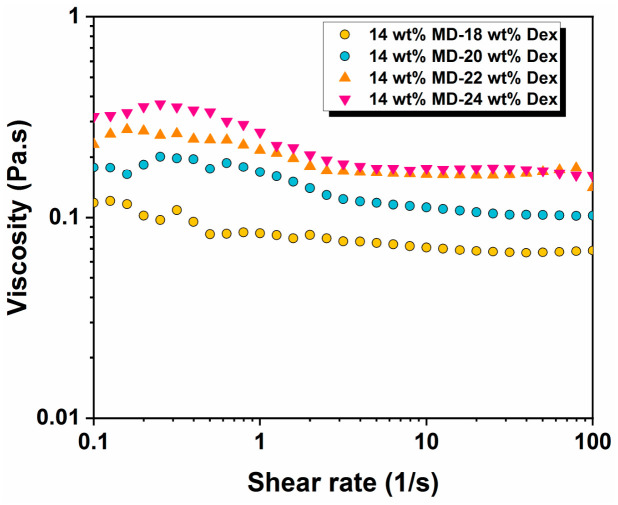
Relationship between emulsion viscosity and shear rate at different concentrations of Dex. The concentrations of BCNCs and MD were fixed at 0.16 wt% and 14 wt%, respectively.

The relationship between viscosity and shear rate for freshly prepared emulsions at different Dex concentrations was also analyzed (Figure 7). Across the shear rate range of 0.1–100 s^−1^, all emulsions showed typical shear-thinning behavior. However, beyond 1 s^−1^ the dependence of viscosity on shear rate diminished, resulting in an apparent Newtonian plateau. This “two-stage” flow profile was consistent with observations from the previous section. Additionally, initial viscosity increased with higher Dex concentrations. According to the Stokes equation, greater viscosity can delay emulsion destabilization within a certain concentration range. Based on these findings, a Dex concentration of 24 wt% was selected as the continuous phase for subsequent experiments.

### 3.5. Contact Angle

**Figure 8:**
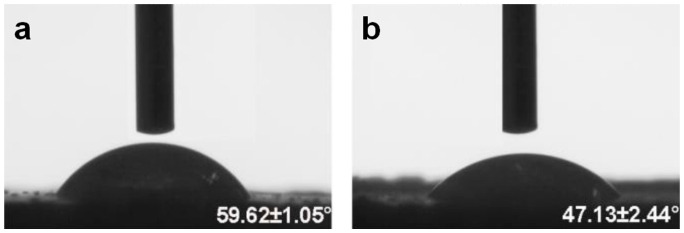
Contact angles of BCNC in the (**a**) MD phase and the (**b**) Dex phase.

Based on the findings described above, the contact angles of BCNCs in the 14 wt% MD phase and the 24 wt% Dex phase were measured to assess their wetting preference. As shown in Figure 8, the contact angle in the MD phase was 59.62 ± 1.05°, while that in the Dex phase was 47.13 ± 2.44°. Both values were below 90°, indicating partial wettability in both phases [44]. However, the lower contact angle in the Dex phase suggests that BCNCs have better wettability and a stronger affinity for this phase, implying a greater tendency to disperse within it. This difference in wettability plays an important role in determining the emulsion’s stability and microstructure. In W/W Pickering emulsions, BCNCs function as interfacial stabilizers, and their adsorption behavior at the phase boundary directly influences droplet size and stability. A lower contact angle in the Dex phase indicated that the BCNCs could be more readily adsorbed at the interface and formed a stable layer, effectively lowering interfacial tension and preventing droplet coalescence and aggregation. This contributes to maintaining the structural integrity of the emulsion and improving its overall physical stability. Wettability differences also affected the rheological behavior of the emulsion. The preferential dispersion of BCNCs in the Dex phase promoted the formation of a more homogeneous network structure, which enhanced viscosity at low shear rates and increased yield stress. Under high shear, this structure responds dynamically, exhibiting pronounced shear-thinning behavior and quickly recovering when the shear is removed. As a result, the emulsion demonstrates excellent processability and mechanical stability.

### 3.6. The Effect of BCNC Concentration on W/W Pickering Emulsions

**Figure 9:**
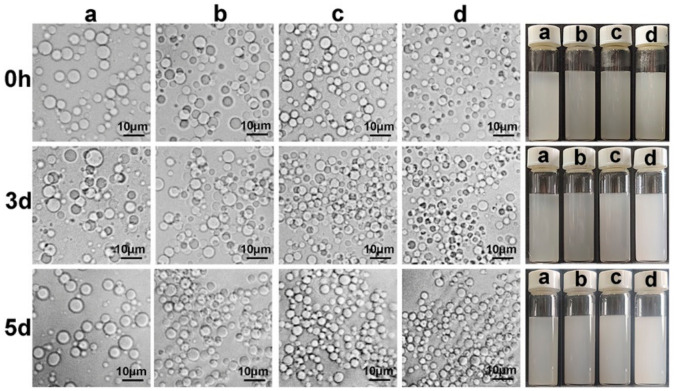
Microscopic morphology and macroscopic phase behavior of W/W Pickering emulsions stabilized by BCNC at different concentrations: (**a**) 0.16 wt%, (**b**) 0.20 wt%, (**c**) 0.24 wt%, and (**d**) 0.28 wt%. The concentrations of MD and Dex were fixed at 14 wt% and 24 wt%, respectively. Scale bar: 10 μm.

The concentration of stabilizers had a significant impact on both the morphology and physical stability of the emulsions. Theoretically, increasing the stabilizer concentration should lead to small emulsion droplets. As shown in Table S3 and Figure 9, the average droplet size of W/W Pickering emulsions decreased with increasing BCNC concentration. Statistical analysis confirmed that, within the range of 0.16 wt% to 0.28 wt%, the average droplet size decreased from 3.3 ± 0.5 μm to 2.8 ± 0.4 μm as the BCNC concentration increased. This phenomenon is likely due to the formation of a denser interfacial network at higher BCNC concentrations, which more effectively restricted the coalescence and aggregation of droplets. From a colloidal interaction standpoint, both attractive and repulsive forces exist between BCNC particles. Generally, repulsive interactions promote emulsion stability, while attractive forces may cause particle aggregation destabilization. As can be seen from Figure 1, after being hydrolyzed with concentrated sulfuric acid, the BC fibers were shortened and functionalized with the negatively charged -SO_3_^−^ group. The uniform surface change increased electrostatic interactions between fibers, thereby improving their emulsifying efficiency.

**Figure 10:**
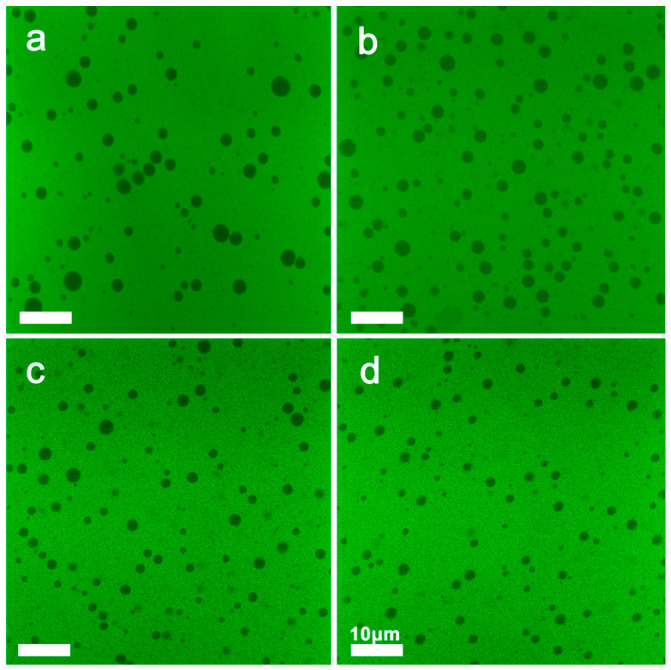
Fluorescence microscopy images of W/W Pickering emulsions stabilized by BCNCs at different concentrations: (**a**) 0.16 wt%, (**b**) 0.20 wt%, (**c**) 0.24 wt%, and (**d**) 0.28 wt%. The black areas represent the MD-rich phase, and the green areas represent the Dex-rich phase. The concentrations of MD and Dex were fixed at 14 wt% and 24 wt%, respectively. Scale bar: 10 μm.

Macroscopically, all freshly prepared emulsions showed no signs of phase separation, which may be attributed to the effective adsorption of BCNC at the water–water interface, providing electrostatic repulsion between BCNCs [23], thereby reducing the Brownian motion of the droplets and consequently inhibiting droplet coalescence. In a study by Nie et al., a W/W emulsion was constructed, with amylopectin serving as the dispersed phase and hydroxypropyl methylcellulose as the continuous phase. The research also explored the storage stability of W/W Pickering emulsions stabilized by varying concentrations of CNC at ambient temperature. The finding revealed that, in contrast to the control group without stabilizer, which exhibited phase separation after 24 h of storage at ambient temperature, the stability of the W/W Pickering emulsions stabilized by different concentrations of CNC was significantly enhanced [18]. A similar phenomenon was also observed in this study. By adjusting the phase ratio and stabilizer concentration, the BCNC-stabilized W/W Pickering emulsions remained stable without phase separation or creaming after being stored at ambient temperature for 5 days. This stands in sharp contrast to emulsions lacking stabilizing particles, which separated after only 1 day under identical conditions. The incorporation of BCNCs significantly enhanced the physical stability of the Dex/MD-based W/W Pickering emulsions. At the microscopic level, the emulsion stabilized with 0.24 wt% BCNC exhibited a more uniform droplet size distribution compared to other formulations. To visualize the phase structure, the Dex-rich phase was stained with FITC-Dex and observed using Fluorescence microscopy. As shown in Figure 10, as the concentration of BCNCs increased from 0.16 wt% to 0.28 wt%, the droplet size of the W/W Pickering emulsion gradually decreased, which is consistent with the results observed by optical microscopy. Furthermore, based on the principle of phase volume, the phase with the larger proportion typically serves as the continuous phase [43]. Therefore, in emulsions where MD and Dex concentrations were 14 wt% and 24 wt%, respectively, the MD-rich phase structure was within the Dex-rich phase.

**Figure 11:**
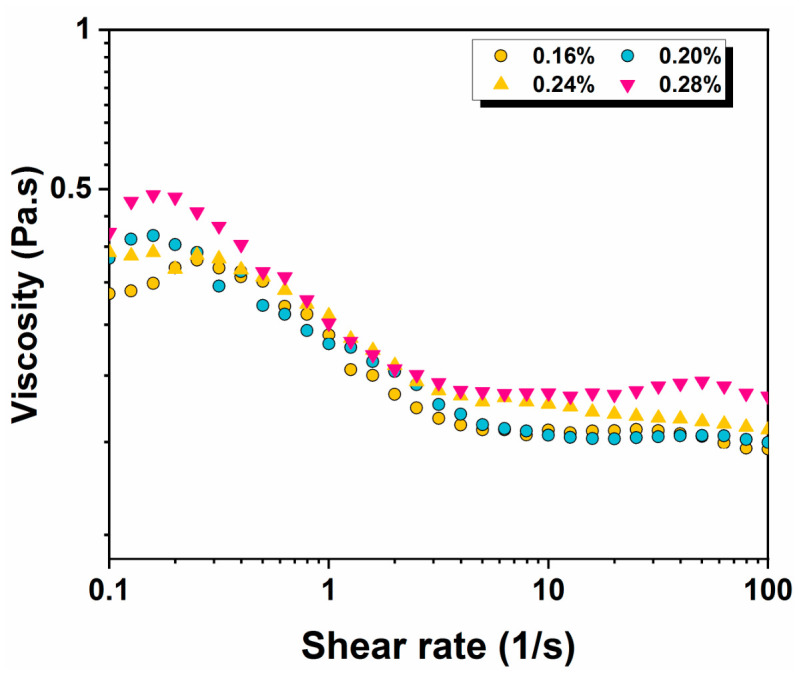
Relationship between the viscosity and shear rate of W/W Pickering emulsions stabilized by BCNCs at different concentrations. The concentrations of MD and Dex were fixed at 14 wt% and 24 wt%, respectively.

The viscosity of BCNC-stabilized W/W Pickering emulsions exhibited shear-thinning behavior, decreasing as the shear rate increased from 0.1 to 1 s^−1^ (Figure 11). With an increase in the concentration of BCNCs, the viscosity of the emulsions also increased, attributed to the more compact network structure of the colloidal solution at higher concentrations. Within the shear rate range of 1–100 s^−1^, viscosity was relatively insensitive to shear rate, suggesting an approximately Newtonian flow behavior. Based on these findings, a BCNC concentration of 0.24 wt% was selected as the optimal stabilizer for subsequent studies.

### 3.7. Effects of NaCl and pH on W/W Pickering Emulsions

**Figure 12:**
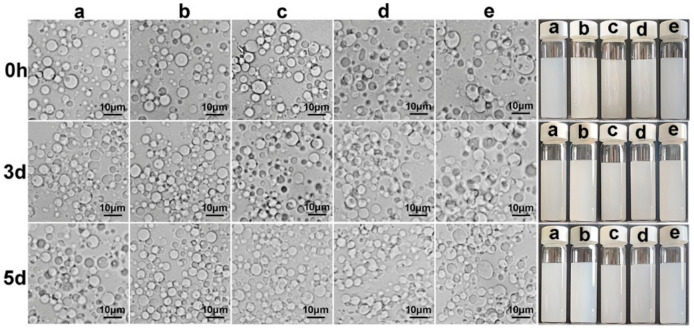
Effects of NaCl concentration on the microstructure and macroscopic phase behavior of W/W Pickering emulsions: (**a**) 0 mM, (**b**) 1 mM, (**c**) 3 mM, (**d**) 5 mM, and (**e**) 7 mM. The concentrations of MD, Dex, and BCNCs were fixed at 14 wt%, 24 wt%, and 0.24 wt%, respectively. Scale bar: 10 μm.

Figure 12 shows the influence of NaCl with varying concentrations of (0, 1, 3, 5, and 7 mM) on the microstructure and storage stability of the emulsions. The W/W Pickering emulsions were highly sensitive to NaCl. Within the tested concentration range, increasing NaCl levels led to noticeable structural deformation. As the concentration of NaCl was increased, a gradual reduction in the number of emulsion droplets was observed in the micro-phase. This was attributed to Na^+^ ions shielding the negatively charged surface of BCNCs, thereby reducing their ζ-potential, weakening electrostatic repulsion, and compromising emulsion stability. This observation contrasts with the findings of Ben Ayed and his co-workers [8], who reported that adding NaCl to poly(ethylene oxide)-dextran W/W emulsions stabilized by CNC induced aggregation of CNC, leading to the formation of a self-supporting gel at a CNC concentration of ≥3 g/L. In this study, rapid aggregation resulted in the formation of an emulsion gel with no creaming when the CNC concentration was higher than 2 g/L. In this study, despite the change in microstructure, no phase separation was observed at the macroscopic level, indicating that the overall integrity of the emulsions remained intact and phase separation was still effectively inhibited.

**Figure 13:**
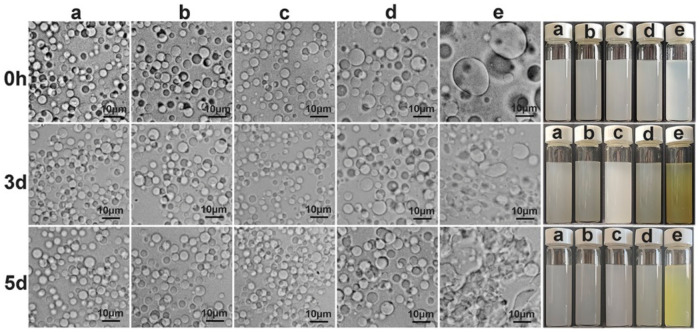
The effect of pH on the microstructure and macroscopic phase behavior of W/W Pickering emulsions: (**a**) 3.0, (**b**) 6.0, (**c**) 7.0, (**d**) 9.0, and (**e**) 11.0. The concentrations of MD, Dex, and BCNCs were fixed at 14 wt%, 24 wt%, and 0.24 wt%, respectively. Scale bar: 10 μm.

To further assess W/W Pickering emulsion stability under different pH conditions, the pH of freshly prepared emulsions was adjusted to 3.0, 6.0, 7.0, 9.0, and 11.0. As shown in Figure 13, the emulsions remained stable at pH 3.0, 6.0, and 7.0, with no significant change in droplet size. However, in alkaline environments (pH 9 and 11), the droplet size was increased markedly with rising pH. At pH in particular, severe deformation was observed; droplets lost their spherical shape, becoming elliptical, and their droplet structure gradually deteriorated during storage. This may be due to the loss of surface-charged groups (e.g., -SO_3_^−^) on the nanocrystals under strongly alkaline conditions, diminishing their ability to stabilize the W/W Pickering emulsions. Additionally, some degree of flocculation occurred in all emulsions during the storage period. At pH 11.0, although no visible phase separation occurred, the emulsions changed in appearance from milky white to yellow, likely due to chemical alterations in the polysaccharide components under high pH.

### 3.8. Effect of AMP on Emulsion Structure and Its Distribution Characteristics

In this section, the concentrations of Dex, MD, and BCNCs were fixed at 24 wt%, 14 wt%, and 0.24 wt%, respectively, and the effects of varying AMP concentrations (0.2–0.8 wt%) on the structure and morphology of W/W Pickering emulsions were investigated (Figure S2). These samples were designated as Dex/MD-BCNC-0.2AMP, Dex/MD-BCNC-0.4AMP, Dex/MD-BCNC-0.6AMP, and Dex/MD-BCNC-0.8AMP, respectively. Concurrently, pure AMP solution (0.6 wt%, 0.6AMP) and emulsion without BCNC stabilization (Dex/MD-0.6AMP) were employed as control groups. The overall morphology and microstructure of the emulsions were characterized using optical microscopy. The results showed that within the AMP concentration range of 0.2–0.8 wt%, all emulsion samples maintained good structural integrity, with no evident morphological deformation, droplet breakup, or coalescence observed (Figure S2c–f). Further droplet size analysis indicated that, compared with the W/W Pickering emulsion without AMP addition, no statistically significant differences in droplet size were detected upon the incorporation of AMP in the range of 0.2–0.8 wt% (*p* > 0.05). This result suggested that, within the investigated concentration range, the introduction of AMP exerted a limited influence on droplet size and did not induce pronounced structural changes in the emulsion system. Meanwhile, to distinguish the intrinsic behavior of AMP from the potential effects arising from the absence of stabilizers, a pure AMP solution with a concentration of 0.6 wt% (Figure S2a) and a W/W emulsion containing 0.6 wt% AMP but without any stabilizer (Figure S2b) were prepared as control samples. Comparative analysis demonstrated that no obvious differences in droplet structure, morphological features, or droplet size distribution were observed between the control samples and the corresponding experimental groups (Table S4).

**Figure 14:**
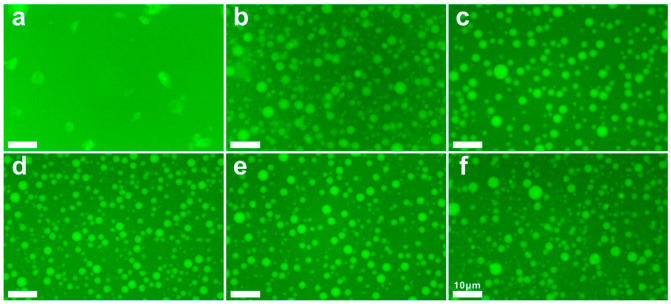
Fluorescence microscopy images of amylopectin (AMP) of different concentrations in solution or W/W emulsions. (**a**) pure AMP solution at 0.6 wt% (0.6AMP); (**b**) W/W emulsion containing 0.6 wt% AMP (Dex/MD-0.6AMP); and (**c**–**f**) W/W Pickering emulsion stabilized by BCNCs with AMP concentrations of (**c**) 0.2, (**d**) 0.4, (**e**) 0.6, and (**f**) 0.8 wt%, respectively (Dex/MD-BCNC-0.2/0.4/0.6/0.8AMP). The concentrations of MD, Dex, and BCNCs were fixed at 14 wt%, 24 wt%, and 0.24 wt%, respectively. Scale bar: 10 μm.

Furthermore, to clarify the partitioning behavior of AMP in the W/W Pickering emulsion, AMP was labeled with FITC fluorescent dye, and the emulsion droplets were imaged using Fluorescence microscopy. As shown in Figure 14, AMP was predominantly distributed within the dispersed phase (MD-rich phase), and the droplet structure, morphology, and size observed in the Fluorescence microscopy images were consistent with those obtained from optical microscopy. The preferential localization of AMP in the MD-rich phase indicated that AMP tended to coexist with MD rather than migrate into the Dex-rich continuous phase or accumulate at the water–water interface. This partitioning behavior was likely associated with the high hydrophilicity of AMP and its thermodynamic compatibility with the polymer-rich phase. In ATPSs, solute partitioning is generally governed by the combined effects of polymer composition, excluded volume effects, and solute–polymer interactions [45]. Under the conditions of this study, AMP, as a highly branched macromolecular polysaccharide, was more prone to remain within the MD-rich dispersed phase, making effective accumulation at the interface unfavorable. Consistently, despite the incorporation of different AMP concentrations, neither the morphological integrity nor the droplet size distribution of the emulsions exhibited significant variations.

### 3.9. In Vitro Digestion

**Figure 15:**
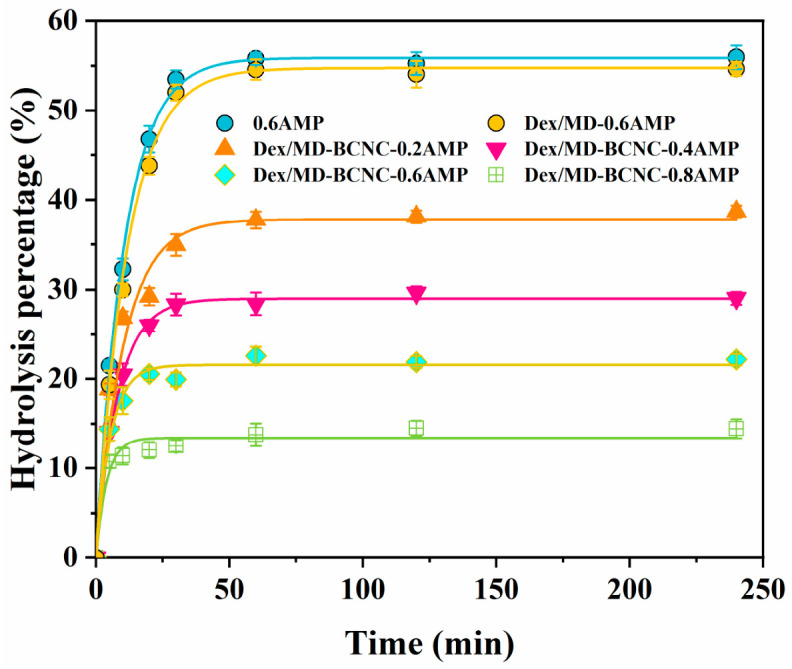
In vitro digestion kinetics of amylopectin (AMP)-based samples in the presence of α-amylase. Sample groups: 0.6AMP, Dex/MD-0.6AMP, Dex/MD-BCNC-0.2/0.4/0.6/0.8AMP. Solid lines represent fitting curves based on the pseudo-first-order kinetic model.

In vitro simulated digestion experiments were conducted for the Dex/MD-BCNC-0.2AMP, Dex/MD-BCNC-0.4AMP, Dex/MD-BCNC-0.6AMP, Dex/MD-BCNC-0.8AMP, 0.6AMP, and Dex/MD-0.6AMP samples in the presence of α-amylase. As shown in Figure 15, the discrete data points represent the experimentally measured values, whereas the solid curves correspond to the predicted profiles obtained from nonlinear fitting based on a pseudo-first-order kinetic model. The digestion kinetics revealed that both the 0.6AMP and Dex/MD-0.6AMP samples underwent rapid hydrolysis characteristics within the initial 30 min, indicating high accessibility of free AMP to α-amylase. These two groups displayed comparable digestive behaviors, reaching similar hydrolysis extents of 55% and 54%, respectively. The BCNC-stabilized Pickering emulsion samples exhibited markedly reduced hydrolysis rates and extents. Specifically, the hydrolysis percentages of Dex/MD-BCNC-0.2AMP, Dex/MD-BCNC-0.4AMP, Dex/MD-BCNC-0.6AMP, and Dex/MD-BCNC-0.8AMP within the first 30 min were approximately 35%, 28%, 19%, and 12%, respectively, with corresponding asymptotic hydrolysis extents ($C_{\infty }$) of 38%, 29%, 22%, and 14%. This pronounced reduction in enzymatic hydrolysis confirms that BCNC-stabilized W/W Pickering emulsions effectively retard AMP digestion via a physical barrier mechanism. In this system, the adsorption of BCNC at the water–water interface restricts the accessibility of amylase to the substrate. In the absence of a stabilizer, direct contact between AMP and digestive enzymes resulted in substantially accelerated hydrolysis. These observations are consistent with previous reports on polysaccharide-based W/W Pickering emulsion systems [18]. While the physical barrier effect imposed by BCNCs at the water–water interface represents the primary mechanism retarding starch hydrolysis, restricted diffusion of α-amylase within the polymer-rich phases and altered substrate accessibility may also contribute to the observed reduction in digestion rates. The progressively lower hydrolysis rates observed with increasing AMP concentration likely reflect an enhanced interfacial packing density of BCNCs, which strengthens the physical barrier, as well as an increased viscosity of the polymer-rich phase that restricts enzyme mobility. Although quantitative discrimination among these contributions is not currently feasible, the dominant role of interfacial BCNC stabilization is supported by the marked contrast between the Pickering emulsion samples and the unstabilized controls. Furthermore, all digestion profiles were well fitted by the pseudo-first-order kinetic model, and the corresponding kinetic parameters are summarized in Table 1. High determination coefficients (R^2^ ≈ 0.99) were obtained for all samples, confirming that the hydrolysis of starch-containing systems under α-amylase catalysis strictly followed pseudo-first-order kinetic behavior [46].

## 4. Conclusions

This study successfully constructed food-grade W/W Pickering emulsions stabilized by BCNCs, using Dex and MD as phase-forming components. The optimized formulation produced emulsions with uniform droplet size and good storage stability over 5 days. Contact angle measurements and microscopic observations suggested that BCNC particles exhibited preferential affinity toward the Dex-rich phase and accumulated at the interface, which was consistent with the observed resistance to coalescence. The emulsions maintained stability across a pH range of 3.0–7.0 but showed sensitivity to ionic strength. Fluorescence imaging revealed that AMP was predominantly localized within the dispersed phase. Digestion kinetic studies further showed that BCNC-stabilized W/W Pickering emulsions significantly reduced the hydrolysis rate of AMP compared to unstabilized controls, a finding consistent with the proposed interfacial barrier effect restricting enzyme-substrate accessibility. Overall, this work presents a facile strategy for constructing stable food-grade W/W Pickering emulsions using natural, biodegradable colloidal particles, with potential applications in functional food systems such as slowly digestible, low glycemic index products.

## Figures and Tables

**Table 1:** Hydrolysis kinetic parameters of AMP-based samples.

Sample	$p_{\infty }$ (%)	*k*	R^2^
0.6AMP	55.91 ± 0.33 ^a^	0.0928	0.9996
Dex/MD-0.6AMP	54.77 ± 0.44 ^a^	0.0852	0.9997
Dex/MD-BCNC-0.2AMP	37.80 ± 0.93 ^b^	0.0963	0.9989
Dex/MD-BCNC-0.4AMP	28.99 ± 0.01 ^c^	0.1214	0.9998
Dex/MD-BCNC-0.6AMP	21.56 ± 0.43 ^d^	0.8195	0.9998
Dex/MD-BCNC-0.8AMP	13.41 ± 0.48 ^e^	0.2498	0.9988

Note: different letters had significant differences at the 5% significance level; the same letter showed that the differences were not significant.

## Data Availability

The original contributions presented in this study are included in the article/Supplementary Material. Further inquiries can be directed to the corresponding authors.

## Supplementary Materials

The following supporting information can be downloaded at: https://www.mdpi.com/article/10.3390/foods15142550/s1.
