# Supplementary material for: Bacterial Cellulose Nanocrystal-Stabilized Water-in-Water Pickering Emulsions: Stability, Amylopectin Partitioning, and In Vitro Digestion Behavior
Source: Foods. 2026 Jul 20;15(14):2550. doi: 10.3390/foods15142550 (PMC13409435; doi:10.3390/foods15142550)
Supplement: Supplementary file 1 [file foods-15-02550-s001.zip › foods-4407214-supplementary.pdf]

Supplementary Material

Figure S1. Phase diagram of MD and Dex. Filled symbols represented the single phase and dashed symbols represented two phases region.

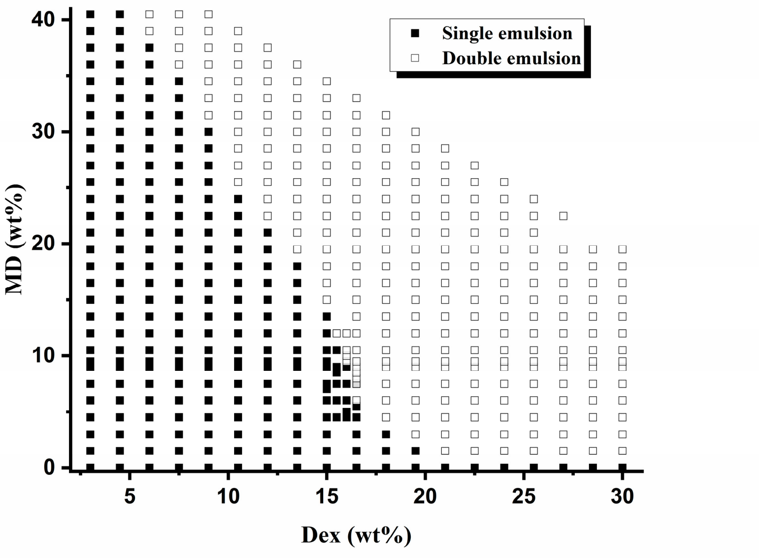

Figure S2. Microstructure of BCNC)-stabilized W/W Pickering emulsions with varying AMP concentrations: (a) 0.2 wt%, (b) 0.4 wt%, (c) 0.6 wt%, and (d) 0.8 wt%. The concentrations of Dex, MD, and BCNC were fixed at 24 wt%, 14 wt%, and 0.24 wt%, respectively. Scale bar was 10 μm.

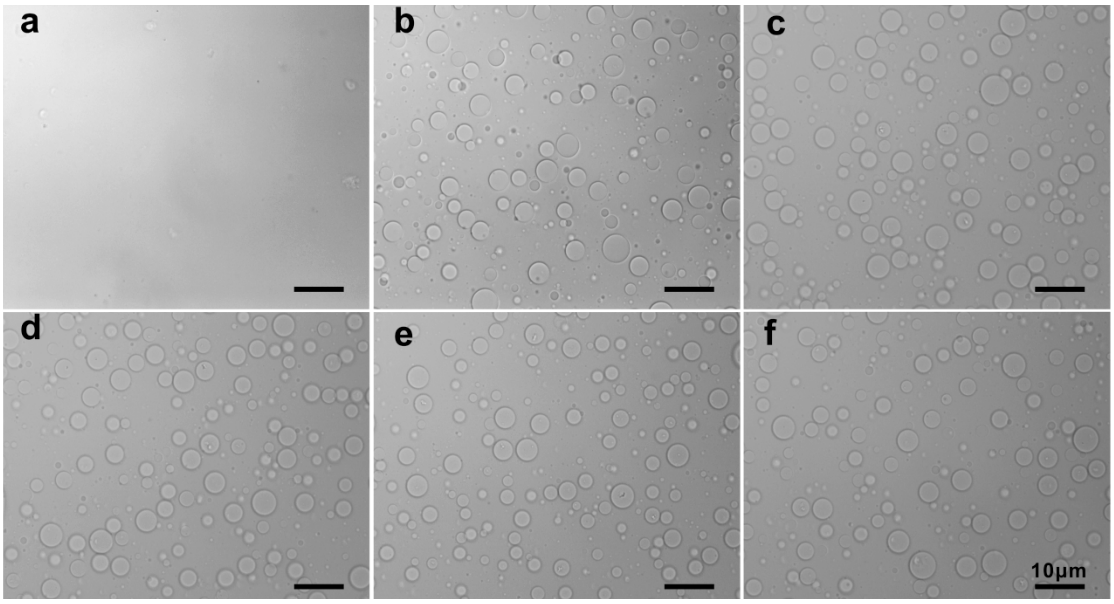

Table S1. The initial average droplet size of W/W Pickering emulsion droplets containing different MD concentrations. The concentrations of Dex and BCNC were fixed at 20 wt% and 0.16 wt%, respectively.

MD concentration (wt%)	The average droplet size (μm)
8	2.3 ± 0.3[d]
10	2.5 ± 0.4[c]
12	3.2 ± 0.5[b]
14	4.0 ± 0.5[a]

Note: different letters had significant differences at the 5% significance level; the same letter showed that the differences were not significant.

Table S2. The initial average droplet size of W/W Pickering emulsion containing different Dex concentrations. The concentrations of MD and BCNC were fixed at 14 wt% and 0.16 wt%, respectively.

Dex concentration (wt%)	The average droplet size (μm)
18	4.6 ± 0.7[a]
20	3.5 ± 0.6[b]
22	3.4 ± 0.5[b]
24	3.2 ± 0.5[c]

Note: different letters had significant differences at the 5% significance level. The same letter showed that the differences were not significant.

Table S3. Initial droplet sizes of W/W Pickering emulsions stabilized by different concentrations of BCNC. The concentrations of MD and Dex were fixed at 14 wt% and 24 wt%, respectively.

BCNCs contents (wt%)	The average droplet size (μm)
0.16	3.3 ± 0.5[a]
0.20	3.3 ± 0.5[a]
0.24	3.0 ± 0.3[b]
0.28	2.8 ± 0.4[c]

Note: different letters had significant differences at the 5% significance level. The same letter showed that the differences were not significant.

Table S4. The influence of AMP concentration on the particle size of the emulsion.

Type	The average droplet size (μm)
0.6AMP	-
Dex/MD-0.6AMP	3.2 ± 0.9^{a}
Dex/MD-BCNC-0.2AMP	3.2 ± 0.7^{a}
Dex/MD-BCNC-0.4AMP	3.1 ± 0.8^{b}
Dex/MD-BCNC-0.6AMP	3.2 ± 0.9^{a}
Dex/MD-BCNC-0.8AMP	3.1 ± 0.8^{b}

Note: The concentrations of Dex, MD, and BCNC were 24 wt%, 14 wt%, and 0.24 wt%, respectively. 0.6AMP: pure AMP solution at 0.6 wt%; Dex/MD-0.6AMP: W/W emulsion containing 0.6 wt% AMP; Dex/MD-BCNC-0.2/0.4/0.6/0.8AMP: W/W Pickering emulsion stabilized by BCNC with AMP concentration of 0.2, 0.4, 0.6, and 0.8 wt%, respectively. Different letters had significant differences at the 5% significance level. The same letter showed that the differences were not significant.
